# Molecular super-gluing: a straightforward tool for antibody labelling and its application to mycotoxin biosensing

**DOI:** 10.1007/s00216-021-03841-3

**Published:** 2022-01-03

**Authors:** Fernando Pradanas-González, Bettina Glahn-Martínez, Elena Benito-Peña, Henri O. Arola, Tarja K. Nevanen, María C. Moreno-Bondi

**Affiliations:** 1 Department of Analytical Chemistry, Faculty of Chemistry, Complutense University of Madrid, Ciudad Universitaria, 28040 Madrid, Spain; 2 VTT Technical Research Centre of Finland Ltd, Tietotie 2, 02150 Espoo, Finland

**Keywords:** SpyTag/SpyCatcher, Fluorescent recombinant fusion proteins, Non-competitive immunoassay, HT-2 toxin, Food safety

## Abstract

Mycotoxins are low molecular weight toxic compounds, which can cause severe health problems in animals and humans. Immunoassays allow rapid, simple and cost-effective screening of mycotoxins. Sandwich assays with a direct readout provide great improvement in terms of selectivity and sensitivity, compared to the widely used competitive assay formats, for the analysis of low molecular weight molecules. In this work, we report a non-competitive fluorescence anti-immune complex (IC) immunoassay, based on the specific recognition of HT-2 toxin with a pair of recombinant antibody fragments, namely antigen-binding fragment (Fab) (anti-HT-2 (10) Fab) and single-chain variable fragment (scFv) (anti-IC HT-2 (10) scFv). The SpyTag and SpyCatcher glue proteins were applied for the first time as a bioconjugation tool for the analysis of mycotoxins. To this aim, a SpyTag-mScarlet-I (fluorescent protein) and scFv-SpyCatcher fusion proteins were constructed, produced and fused in situ during the assay by spontaneous Tag-Catcher binding. The assay showed an excellent sensitivity with an EC50 of 4.8 ± 0.4 ng mL^−1^ and a dynamic range from 1.7 ± 0.3 to 13 ± 2 ng mL^−1^, an inter-day reproducibility of 8.5% and a high selectivity towards HT-2 toxin without cross-reactivity with other *Fusarium* toxins. The bioassay was applied to the analysis of the toxin in an oat reference material and in oat samples, with a LOD of 0.6 µg kg^−1^, and the results were validated by analysing a certificate reference material and by HPLC–MS/MS.

**Graphical abstract:**

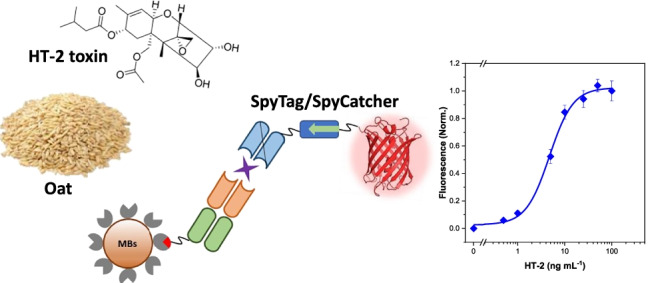

**Supplementary Information:**

The online version contains supplementary material available at 10.1007/s00216-021-03841-3.

## Introduction

Mycotoxins are toxic compounds produced naturally by a wide variety of filamentous fungal species. The main mycotoxin-producing moulds include those of the genus *Aspergillus*, *Fusarium*, *Penicillium*, *Aspergillus, Alternaria* and *Claviceps*, which infect a wide variety of food crops. The Food and Agriculture Organization (FAO) has estimated that more than 25% of the crops around the world have been affected by mycotoxins and recent studies revealed that their occurrence above detectable levels is up to 60–80%. Climate change and the availability of increasingly sensitive analytical methods have been suggested to explain such high occurrence [1].

The presence of these harmful compounds has been reported in different cereals and cereal products on a global scale. Oats are one of the most widely consumed cereals worldwide due to their nutritional properties. They are an important source of fibre, antioxidant compounds (i.e. tocopherols, phenolic compounds, sterols), B vitamins and essential unsaturated fatty acids. In addition, oat consumption has been associated with reduced risk of cardiovascular disease and prevention of other diseases, such as cancer and diabetes [2]. However, the consumption of oats may pose a health risk due to the susceptibility of these crops to mycotoxin contamination. More than 40 different mycotoxins have been found in oats with HT-2 and T-2 toxins being the most commonly reported [3]. These mycotoxins belonging to type A trichothecenes are produced by different *Fusarium* fungal species (e.g. *langsethiae*, *poae*, *sporotrichioides*) and are considered highly toxic [3]. HT-2 and T-2 toxins are structurally very similar and differ only in the C-4 position of the structure, where HT-2 has a hydroxyl group and T-2 an acetate group. In fact, T-2 is rapidly metabolised to an HT-2 toxin after ingestion, and both toxins are considered to be equally toxic. The EU has established recommended values for the sum of both toxins in different foodstuffs [4]. Despite the similarity of their structures and current analysis recommendations, it is interesting to control the level of each mycotoxin in foodstuffs as there are pieces of evidence of their different bioaccessibility [5] and impact on human cells [6].

Mycotoxins are commonly analysed by liquid and gas chromatography coupled to mass spectrometry detectors [7]. Although these techniques are known to be very accurate and sensitive, they have several drawbacks, e.g. high cost of instrumentation and maintenance, need for skilled personnel and laborious sample treatment procedures [8]. In this regard, immunoassays can be a cheaper, simpler and faster alternative for point-of-action applications. Most immunoassays available for HT-2 toxin analysis are based on the competitive assay format, in which the toxin is conjugated to different labels (i.e. fluorophore, enzyme). Chemical synthesis of toxin-conjugates poses a risk for the operator due to the handling of toxic substances. Moreover, conjugations are time-consuming, and the reaction may suffer from high batch-to-batch variations, which will affect the reproducibility of the bioassay. In addition, labelling may modify the toxin structure and its affinity for the antibody compared to the free toxin [9–12]. The implementation of non-competitive immunoassays based on a sandwich format avoids the use of toxin-conjugates for analyte detection and usually provides higher selectivity and, in many cases, sensitivity than competitive assays [13]. However, the development of these assays for mycotoxin analysis is a big challenge as the small size of these molecules does not allow binding of two antibodies simultaneously. One alternative for the implementation of direct readout systems is anti-immune complex-based assays, where a primary antibody binds to the epitope of the target analyte and the resulting immune complex is recognised by a secondary antibody that must be labelled previously [13]. The application of recombinant antibody technology allows the production of secondary antibodies fused to reporter proteins without affecting the antigen-binding site during the derivatization reaction, generally leading to improved sensitivity, although they are not widely applied yet [14].

In this work, we report the development of a non-competitive fluorescence immunoassay for the specific detection of HT-2 toxin in oat samples. The bioassay is based on the application of a pair of recombinant antibodies obtained by phage display [15], namely a primary antigen-binding fragment (Fab), specific to both HT-2 and T-2 toxins (anti-HT-2 (10) Fab), and a secondary single-chain variable antibody fragment which recognises the immune complex formed by Fab and the HT-2 toxin (anti-IC HT-2 (10) scFv). Direct detection of the analyte is achieved by measuring the emission of a fluorescent protein bioconjugated to the assay-complex using the SpyTag and SpyCatcher ligation system. These proteins have been engineered from extracellular proteins of Gram-positive bacteria (*Streptococcus pyogenes*), in which the fibronectin-binding protein (FbaB) was found to be stabilised by spontaneous intramolecular isopeptide bonds. The binding event takes place between two amino acid residues, aspartic acid and lysine, present in the sequence of the SpyTag peptide and in the SpyCatcher protein, respectively, forming a fast and robust irreversible covalent bond over a wide range of temperature, pH and buffers [16]. There are other conjugation techniques, such as SNAP-tag [17] and HaloTag systems [18], which allow the binding of proteins by spontaneous formation of covalent bonds. However, they require the presence of specific organic ligands that must be added to the desired protein by laborious chemical conjugation processes in order to be bound to the fusion proteins formed with the SNAP-tag or HaloTag proteins. The SpyTag/SpyCatcher system allows the heterologous production of SpyTag-proteins and SpyCatcher-proteins followed by a post-translational bioconjugation. This ligation system has been used in different applications, such as cell imaging and microscopy [19], immunology [20], protein-polymer hybrid hydrogels [21] or catalysis [22]; but, so far, its application to the development of immunoassays is scarce [23, 24], and, to the best of our knowledge, it has never been applied to the bioconjugation of antibodies and luminescent proteins for the optical determination of mycotoxins. In this work, we report the application of these “glue proteins” for the generation of the analytical signal upon spontaneous binding of the SpyTag-mScarlet-I fusion protein with the anti-IC HT-2 (10) scFv-Spy Catcher through the formation of an isopeptide bond Tag-Catcher.

## Materials and methods

### Materials and reagents

HT-2 toxin, T-2 toxin, fumonisin B_1_ (FB_1_) and fumonisin B_2_ (FB_2_) were purchased from Fermentek Ltd (Jerusalem, Israel), deoxynivalenol (DON) and zearalenone (ZEA) and PBS powder from Sigma-Aldrich (St. Louis, MO, USA). Antibody fragment (anti-HT-2 (10) Fab) and sequence coding for single-chain variable antibody fragment (anti-IC HT-2 (10) scFv) were provided by VTT Technical Research Centre (Espoo, Finland). Plasmids pZsYellow and pmCherry were acquired from Clontech (Mountain View, CA, USA), pQE-T7-2 together with QIAquick PCR clean-up kit and QIAprep spin mini-prep kit from Qiagen (Hilden, Germany), pmScarlet-I from Instituto de Salus Carlos III (Majadahonda, Madrid, Spain), pTagRFP and *E*. *coli* (DE3) pLysS from CIB (Madrid, Spain) and pMAL-C5X, NEBuilder HiFi DNA Assembly Master Mix, *E*. *coli* NEB 5α and Shuffle Express Competent *E. coli* from New England biolabs (Ipswich, MA, USA). Phusion Hot Start II HF DNA polymerase, EZ-link sulfo NHS-LC-LC-Biotin, Pierce™ Protein Free (PP Free) blocking buffer and Superblock™ (SBlock) blocking buffer in PBS and 96 flat-bottom well plates were obtained from Thermo Scientific (Waltham, MA, USA). Bacterial cell lysis buffer and Luria Broth (LB) medium were obtained from NZYTech (Lisbon, Portugal) and HisTrap FF crude, PD-10 and illustra NAP 5 columns were purchased from GE Healthcare (Chicago, IL, USA). Kaivogen Kaisa96 streptavidin plates were acquired from Kaivogen (Turku, Finland) and SpeedBeads magnetic neutravidin-coated particles from Cytiva (Marlborough, MA, USA). All primers and plasmids containing the genes encoding for SpyCatcher and anti-IC HT-2 (10) scFv were bought from Integrated DNA Technologies (Coralville, IA, USA). Finally, oat reference material was bought from Aokin (Berlin, Germany), and blank oat sample and naturally contaminated oat were provided by Prof. B. Patiño from the Faculty of Biology of the University Complutense of Madrid (Madrid, Spain).

### Methods

#### Antibody fragment (Fab) biotinylation

Anti-HT-2 (10) Fab was obtained from the phage display antibody library as reported previously [15]. According to the manufacturer’s instructions, the Fab fragment was incubated with a 20-fold molar excess of activated biotin reagent during 30 min at room temperature. The biotin excess was removed with an exclusion molecular column (NAP-5 columns), and the biotinylated Fab (bio-Fab) was eluted with PBS (0.01 M phosphate-buffered saline, NaCl 0.138 M, KCl 2.7 mM, pH 7.4).

#### Construction, expression and purification of the recombinant fusion proteins

The SpyTag peptide was genetically fused to different fluorescent proteins (FP: mScarlet-I, TagRFP, mCherry and ZsYellow), and the SpyCatcher protein was fused to the single-chain variable antibody fragment anti-IC HT-2 (10) scFv, using standard molecular biology techniques [25]. Different expression vectors and *E. coli* cells were used depending on the fusion protein features.

**Fig. 1:**
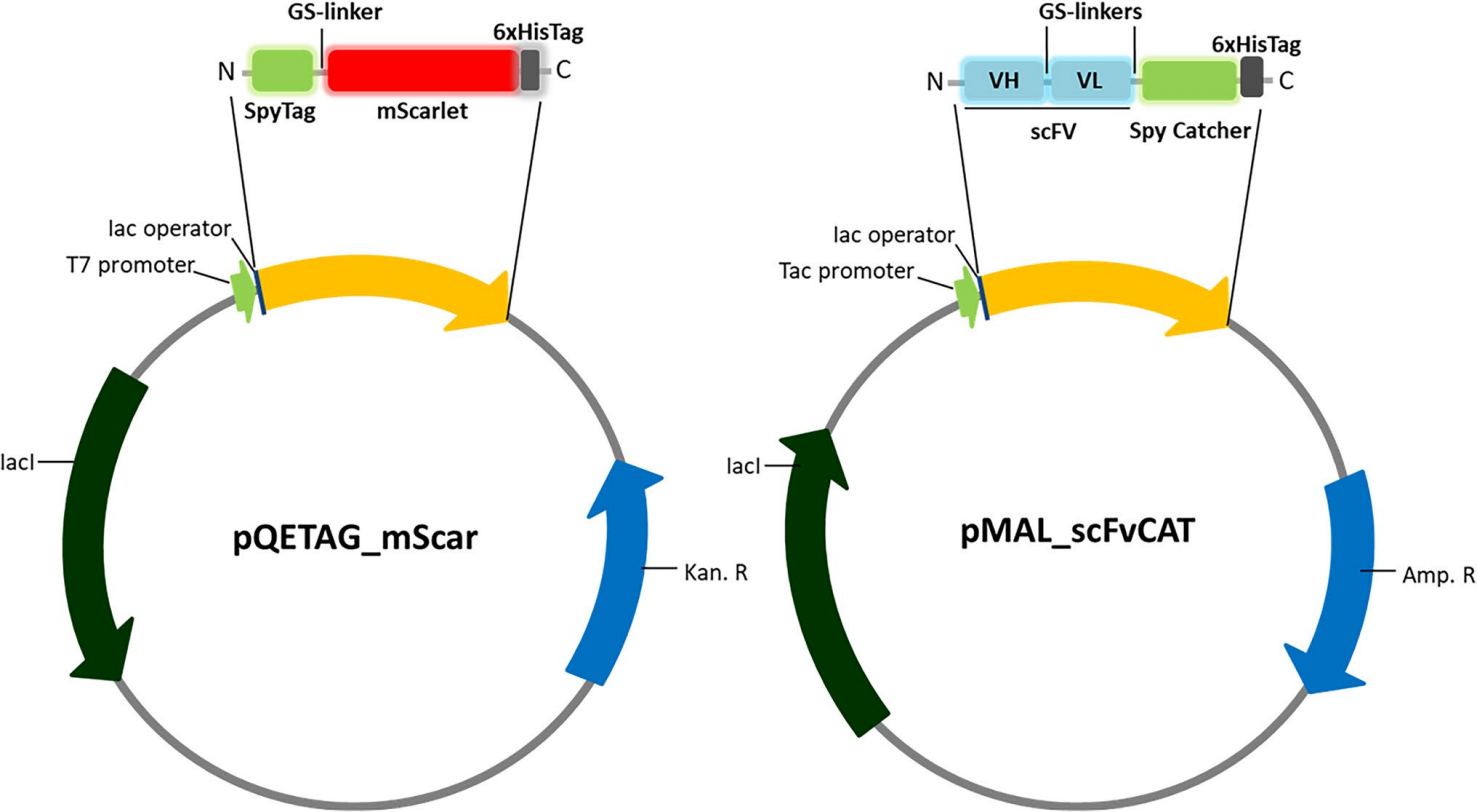
Map of the fusion protein constructs and main characteristics of expression vectors used for the production of the recombinant proteins in different E. coli strains. SpyTag-mScarlet-I in pQE-T7-2 (left side) and anti-IC HT-2 (10) scFv-SpyCatcher in pMAL-C5X (right side)

The expression vector pQE-T7-2 was modified to contain the DNA sequence encoding for the SpyTag-mScarlet-I fusion protein (Fig. 1) to create the plasmid pQETAG_mScar (pQETAG_FP, in general). The mScarlet-I gene was amplified using the polymerase chain reaction (PCR) by Phusion HF DNA polymerase from the vector pmScarlet-I with the primers FwP_SpyTag_mS and RP_SpyTag_mS (Supplementary Material Table S1). The vector pQE-T7-2 was also amplified with the oligonucleotides FwP_SpyTag_pQE and RP_SpyTag_pQE (Supplementary Material Table S1). All primers had a sequence for hybridisation with the complementary part of their DNA template and a tail to create overlapping areas between both PCR products, as well as to add different features. Thus, the sequence encoding for the SpyTag peptide was added in the tails of the primers FwP_SpyTag_mS and RP_SpyTag_pQE, and the sequence encoding for a glycine-serine (GS)-linker, to separate the peptide and the fluorescent protein in the primer FwP_SpyTag_mS.

The same methodology was employed to construct the fusion proteins SpyTag-TagRFP, SpyTag-mCherry and SpyTag-ZsYellow, building plasmids pQETAG_TRFP, pQETAG_mChe and pQETAG_ZsY (pQETAG_FP, in general), respectively (Supplementary Material Figure S1 and Table S1).

The DNA sequence of the fusion protein anti-IC HT-2 (10) scFv-SpyCatcher was introduced in the expression vector pMAL-C5X to create the plasmid pMAL_scFvCAT (Fig. 1). The scFv antibody and SpyCatcher genes were amplified from their individual plasmids by PCR, using the primers FwP_scFv/RP_scFv and FwP_SpyCat/RP_SpyCat, respectively (Supplementary Material Table S1). The vector pMAL-C5X was amplified with the oligonucleotides FwP_pMAL and RP_pMAL (Supplementary Material Table S1), creating overlap areas with the above PCR products.

PCR products were purified with the QIAquick PCR clean-up kit and assembled through the DNA overlap areas included in the primers with NEBuilder HiFi DNA assembly Master Mix. The resulting plasmids were transformed into *E. coli* NEB 5α cells and selected on LB agar plates, in the presence of 50 µg mL^−1^ kanamycin for plasmids pQETAG_FP or with 100 µg mL^−1^ ampicillin for the plasmid pMAL_scFvCAT. Individual colonies were picked and grown in LB medium culture during 16 h at 37 °C. Plasmids were extracted and purified with the QIAprep spin mini-prep kit, and successful cloning was confirmed by DNA sequence analysis.

Plasmids pQETAG_FP were transformed into *E. coli* BL21 (DE3) pLysS cells and selected on LB agar plates with 50 µg mL^−1^ kanamycin and 33 µg mL^−1^ chloramphenicol, and the plasmid pMAL_scFvCAT into *E. coli* Shuffle express cells, and selected with 100 µg mL^−1^ ampicillin for the over-expression of the fusion proteins.

Single colonies containing plasmids pQETAG_FP or pMAL_scFvCAT were individually picked and used to inoculate 10 mL of preculture (LB medium supplemented with 50 µg mL^−1^ kanamycin and 33 µg mL^−1^ chloramphenicol for pQETAG_FP or with 100 µg mL^−1^ ampicillin for pMAL_scFvCAT), and they were grown overnight at 37 °C (pQETAG_FP) or 30 °C (pMAL_scFvCAT) with shaking. The next day, the main cultures of 100 mL (LB medium with appropriate antibiotics) were inoculated with each preculture in the exponential growth to an optical density at 600 nm (OD_600_) of 0.05. The cell growth was spectrophotometrically monitored, and they continued growing with shaking at 37/30 °C until an OD_600_ of 0.5–0.6 was reached. Isopropylβ-D-1-thiogalactopyranoside (IPTG) was added to a final concentration of 1 mM (pQETAG_FP in *E. coli* BL21 (DE3) pLysS cells) or 0.4 mM (pMAL_scFvCAT in *E. coli* Shuffle express cells) to induce the expression of the fusion proteins. The cultures continued to grow for 16 h with shaking at 26 °C and 30 °C for the expression of SpyTag-fluorescent protein and scFv-SpyCatcher, respectively. After that, the cells were harvested by centrifugation for 10 min at 5000* g* at 4 °C and resuspended in 5 mL of lysis buffer, per gram of cell paste, supplemented with lysozyme, DNase and protease inhibitor cocktail. The cells were lysed by incubation for 30 min at RT and probe-type sonication on ice for 5 min with 10-s pulses of sonication and break. The remaining cell debris was removed by centrifugation for 15 min at 15,000* g* at 4 °C.

The fusion proteins were purified from the clarified lysate by HisTrap columns according to the manufacturer’s instructions, utilising the 6xHis-tag present in the C-terminus of each recombinant protein, The cell lysate was diluted (1:3 v/v) in the binding buffer (20 mM sodium phosphate, 500 mM NaCl, 20 mM imidazole, pH 7.4) before loading the sample, in order to obtain the optimal pH and ensure protein binding to the column. After sample loading, the column was washed with 20 mL of binding buffer, and the fusion protein was eluted from the column with elution buffer (20 mM sodium phosphate, 500 mM NaCl, 500 mM imidazole, pH 7.4). The presence and purity of each fusion protein in the elution fractions were checked by SDS polyacrylamide gel electrophoresis (PAGE), and the fusion protein–containing fractions were pooled. The buffer was exchanged to PBS, with PD-10 desalting columns according to the manufacturer’s instructions. The concentration of the stock was determined spectrophotometrically with a UV–Vis spectrophotometer NanoDrop one (ThermoFisher, Waltham, MA, USA).

#### Fusion protein characterisation

The fluorescent fusion proteins were characterised spectroscopically in order to study the influence of the added SpyTag peptide at the N-terminus of the fluorescent proteins. Excitation and emission spectra were registered with a spectrofluorometer (FluoroSENS 9003, Gilden photonics). The emission quantum yield was calculated applying the method of Parker-Rees, which is based on the comparison of the fluorescence of the sample with a reference standard whose quantum yield is known and has been determined by an absolute method [26]. Rhodamine 101 (for SpyTag-mScarlet, SpyTag-TagRFP and SpyTag-mCherry) and rhodamine 6G (for SpyTag-ZsYellow) were used as reference fluorophores, since these compounds exhibit excitation and emission spectra similar to those of the recombinant proteins. The experimental quantum yield of the samples was calculated according to Eq. 1:

1 $${\phi }_{f}^{x}= {\phi }_{f}^{s} \left[\frac{{I}_{x}}{{I}_{s}}\right]\left[\frac{{A}_{s}}{{A}_{x}}\right]\left[\frac{{n}_{x}}{{n}_{s}}\right]$$

where ${\phi }_{f}^{s}$ is the quantum yield of the reference fluorophore, *x*, the sample, *s*, the reference, *I,* the integral fluorescence of the emission spectrum, *A*, the absorbance at the excitation wavelength (typically *A* < 0.1) and *n*, the refractive indices of the solvents.

The fluorescence lifetime measurement was performed with an Edinburgh Instruments FLS 980 spectrometer (Livingston, UK), equipped with a 463-nm laser diode pulsed at 500 kHz and a 460-nm band-pass filter for the excitation of the sample. The emission was monitored at the wavelength of the maximum emission of each fusion protein by a blazed double monochromator of 500 nm with a Hamamatsu R928P photomultiplier tube.

Spontaneous binding of the glue proteins SpyTag and SpyCatcher was evaluated by incubating the SpyTag-mScarlet-I and scFv-SpyCatcher fusion proteins in a 1:1 molar ratio at ambient temperature for 30 min. The binding event was confirmed by SDS-PAGE by comparing it to the unreacted fusion proteins.

#### Non-competitive magnetic bead fluorescence immunoassay

**Fig. 2:**
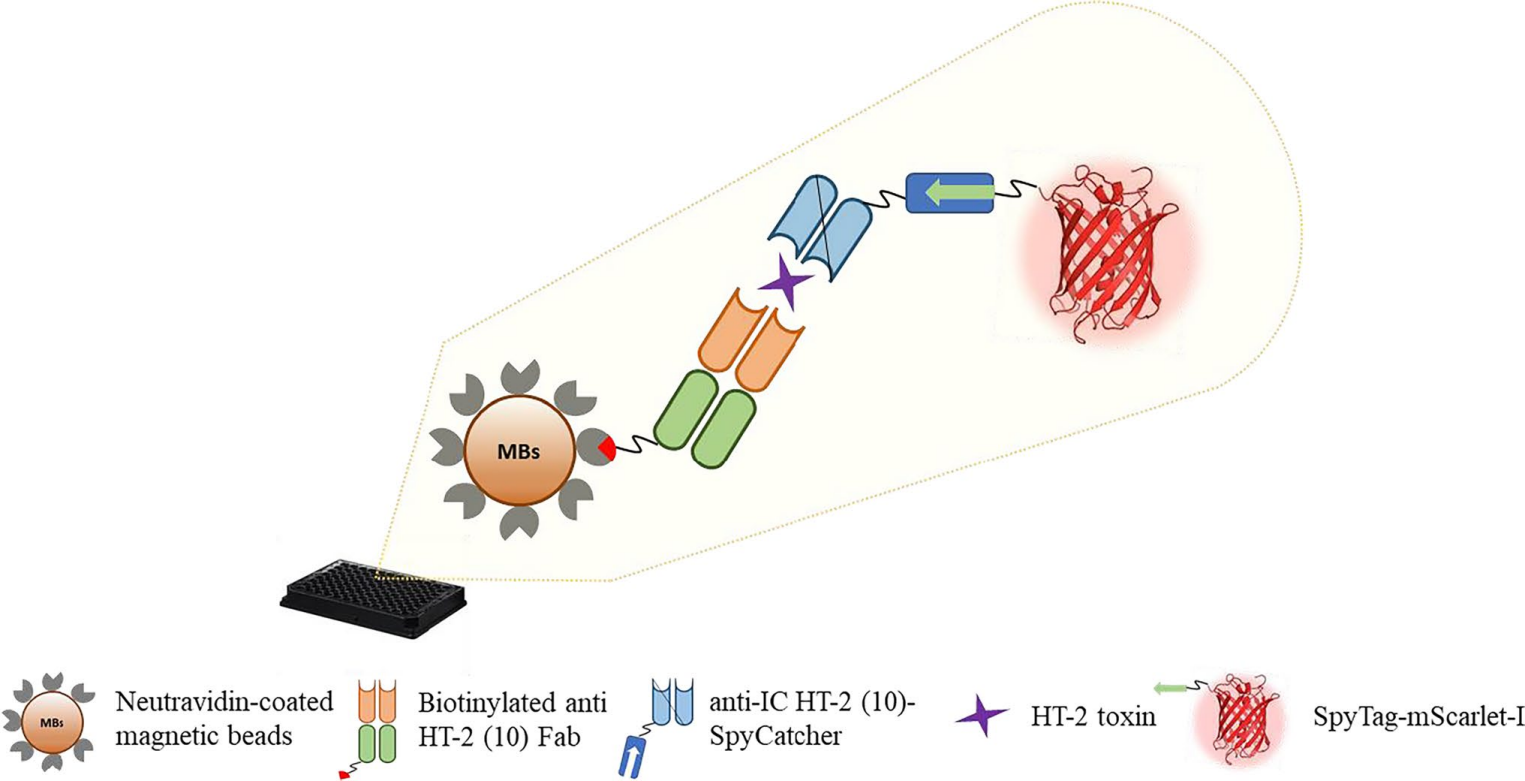
Schematic representation of the non-competitive fluorescence immunoassay for the analysis of HT-2 toxin

The non-competitive fluorescence immunoassays (Fig. 2) were performed in black 96-well plates. Neutravidin-coated magnetic beads (5 µg in 20 µL of PP Free blocking buffer in PBS pH 7.4) and 500 ng of the biotinylated anti-HT-2 (10) Fab (60 µL in PP Free blocking buffer in PBS pH 7.4) were incubated for 30 min with shaking. The wells were washed three times with PBS-T (PBS pH 7.4; 0.05% (v/v) Tween-20) in an automatic magnetic washer (Hydroflex, Tecan, Männedorf, Switzerland). The sample (i.e. HT-2 toxin standards or real sample extracts) and 1000 ng of anti-IC HT-2 (10) scFv-SpyCatcher were added in a total volume of 60 µL (PP Free blocking buffer) and incubated for 30 min with shaking (A). Afterwards, the wells were washed again three times with PBS-T and then incubated with 60 µL of the probe SpyTag-mScarlet-I (1 µM in PP Free blocking buffer) for 15 min with shaking (B). A final washing step (3 × PBS-T) was carried out to remove the excess recombinant fluorescent protein. Finally, 50 µL of PBS was added to each well, and the fluorescence signals were measured with a BMG Labtech CLARIOstar microplate reader (excitation at 569 nm and emission at 593 nm).

#### Sample preparation

Oat samples were homogenised by crushing with a mixer mill MM 200 (from Retsch, Germany) and passed through a 1-mm pore size sieve. A solid–liquid extraction was performed to extract the HT-2 toxin. For this purpose, 1 g of homogenised sample was weighed and extracted with 5 mL of a methanol–water (70:30, v/v) mixture on a shaker (IKA KS 400 i control) for 1 h at room temperature and at 300 rpm. Samples were centrifuged 10 min at 6000* g*, and 1 mL of the supernatant was pre-concentrated tenfold in a Savant DNA speed vac. The final extract was diluted 1:5 in PP Free blocking buffer, in order to avoid the matrix effect, in the presence of the recombinant scFv-SpyCatcher protein.

#### Data analysis

Calibration plots were carried out with the fluorescence signals normalised to the minimum and the maximum signals following Eq. 2 and analysed with Origin Pro 2019 software (OriginLab Corp. Northampton, MA, USA) using a four-parameter logistic regression function (Eq. 3).

2 $${y}_{norm.}= \frac{\left(y-{y}_{min}\right)}{{y}_{max}-{y}_{min}}$$

3 $${y}_{norm.}= {A}_{\mathrm{min}+}\frac{\left({A}_{max}-{A}_{min}\right)}{1+{\left(\frac{x}{{EC}_{50}}\right)}^{b}}$$

where ${A}_{min}$ and ${A}_{max}$ are the asymptotic minimum and maximum, respectively, *b* is the slope of the curve at the inflection point and ${EC}_{50}$ is the HT-2 toxin concentration at the inflection point of the curve. The dynamic range of the immunoassay was established as the concentrations in the 20–80% range of the fluorescence signal (EC_20_ and EC_80_). The limit of detection (LOD) was calculated as the average blank signal + 3 × the standard deviation of the blank.

## Results and discussion

### Construction and characterisation of the fusion proteins

In the first step, we carried out the preparation of the fusion proteins, SpyTag-mScarlet-I and scFv-SpyCatcher by molecular engineering. Construction of such fusions, which can be produced recombinantly in bacteria, allows the development of immunoassays without the need of using secondary antibodies. In addition, the availability of SpyTags fused to different fluorescent proteins facilitates the selection of the best one for a given application and could be used in combination with antibody fragments, fused to the SpyCatcher, selective to different analytes enabling multiplexing. The fusion protein constructs, pQETAG/mScar and pMAL/scFvCAT (Fig. 1), were confirmed by DNA sequence analysis. The correct size of the purified fusion proteins was verified by SDS-PAGE. In order to evaluate if the binding to mScarlet-I or scFv affected the formation of the spontaneous isopeptide bond between the SpyTag and the SpyCatcher, solutions of SpyTag-mScarlet-I and scFv-SpyCatcher were mixed in a 1:1 molar ratio and incubated for 30 min with shaking at RT. The success of ligation was confirmed by SDS-PAGE (Supplementary Material Figure S2). A new band with a molecular weight of *c.a.* 70 kDa, corresponding to the sum of SpyTag-mScarlet-I (30 kDa) and scFv-SpyCatcher (38 kDa), was observed in the gel, confirming successful linkage. Therefore, the use of a GS-linker between SpyTag and mScarlet-I and between scFv and SpyCatcher was enough to achieve the correct folding of each protein and to maintain their functionalities.

The excitation and emission spectra of the SpyTag-mScarlet-I fusion protein were equivalent to the spectra of the reference mScarlet-I (Supplementary Material Figure S3). Moreover, the fluorescence quantum yield (0.34) and the fluorescence emission lifetime (3.72 ns) obtained for the SpyTag-mScarlet-I fusion protein (Supplementary Material Figure S4) were comparable with those reported in the literature for the fluorescent protein [27, 28], confirming that fusion with the SpyTag peptide does not affect its photochemical properties. The excitation and emission spectra of the other SpyTag-FP fusion proteins (FP: TagRFP, mCherry and ZsYellow) (Supplementary Material Figure S3) also showed similar photochemical properties to the respective fluorescent proteins. The fluorescence quantum yield and the fluorescence emission lifetime of the fusion proteins are shown in the Supplementary Material (Figure S4).

### Assay optimisation

Streptavidin-coated 96-well plates (SA plates) and neutravidin-coated magnetic beads (NA-MBs) were tested for the immobilisation of the primary antibody (biotinylated anti-HT-2 (10) Fab). Better signal-to-background (S/B) ratios were obtained using NA-MBs for the assay, probably due to the higher surface-to-volume ratio of the NPs in comparison to the well plates [13]. Different assay buffers (i.e. PBS, PP Free in PBS and SBlock in PBS) were also evaluated for assay optimisation and the best S/B ratios were obtained using the PP Free buffer (PBS, pH 7.4) that was selected for further experiments in combination with NA-MBs (Supplementary Material Figure S5). PP Free blocking buffer contains no protein while SBlock contains a single purified glycoprotein according to the manufacturer. The absence of protein in the PP Free buffer, also according to the manufacturer, minimizes the cross-reactivity and interference problems associated with traditional protein-based blocking buffers as confirmed in the experiment.

A checkerboard titration was carried out to select the optimal concentration of the primary and secondary antibody for the detection of HT-2 toxin. Three concentrations of biotinylated anti-HT-2 (10) Fab (bio-Fab) (300 ng, 500 ng, 700 ng) diluted in 60 μL of PP Free blocking buffer were immobilised on 5 μg of magnetic microbeads (20 μL) in a 96-well plate. The particles were mixed with different concentrations of anti-IC HT-2 (10) scFv-SpyCatcher (350 ng, 700 ng, 1000 ng) diluted in 60 μL of PP Free blocking buffer (Supplementary Material Figure S6). The highest sensitivity was obtained using 500 ng/well of bio-Fab and 1000 ng/well of scFv-SpyCatcher. As it can be observed in Figure S6, higher amounts of scFv-SpyCatcher resulted in higher S/B ratios for a constant concentration of bio-Fab. However, the use of higher amounts of scFv-SpyCatcher (1500 ng/well) in combination with the optimal bio-Fab concentration did not result in an improvement in sensitivity, probably because the binding sites were already saturated. Therefore, neutravidin-coated magnetic beads functionalised with 500 ng of bio-Fab and 1000 ng of scFv-SpyCatcher were selected for further assays.

**Fig. 3:**
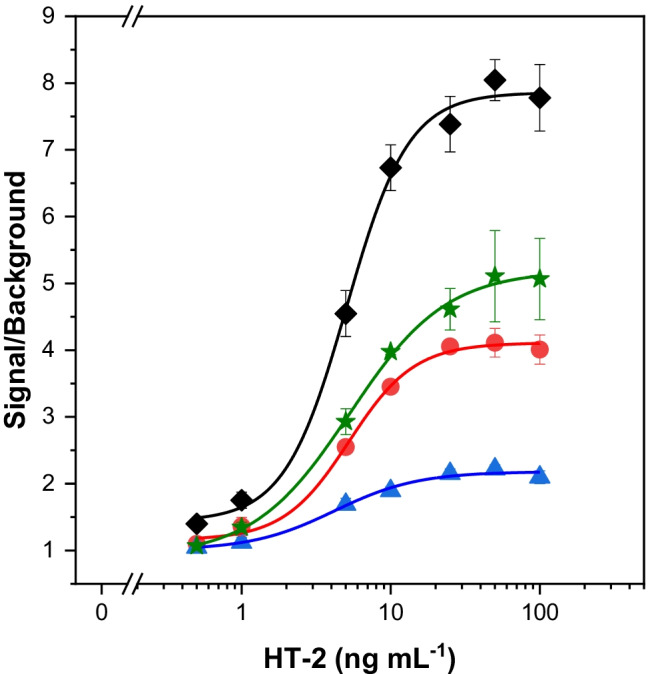
Calibration plots for HT-2 toxin with different fluorescent fusion proteins: SpyTag-mScarlet-I (λexc = 569 nm; λem = 593 nm) (black diamonds), SpyTag-TagRFP (λexc = 543 nm; λem = 585 nm) (red circles), SpyTag-mCherry (λexc = 567 nm; λem = 610 nm) (blue triangles) and SpyTag-ZsYellow (λexc = 500 nm; λem = 545 nm) (green stars). Signal-to-background (S/B) ratios were calculated as the ratio of the fluorescence in the presence of HT-2 toxin and the signal in the absence of the toxin. The results are depicted as S/B ratio means ± the standard error of the mean (n = 3)

Four fluorescent proteins (FP: mScarlet-I, TagRFP and mCherry, ZsYellow), emitting in different regions of the visible spectrum, were tagged with the SpyTag peptide to select the optimal combination for assay development. One of the advantages of using the SpyTag/SpyCatcher system is that it is not necessary to prepare the fusion proteins of the scFv fragment with each fluorescent protein, reducing the synthetic effort of the assay. Moreover, once they are prepared, the SpyTag-FPs can be used in combination with any antibody bound to the SpyCatcher. The calibration curves for HT-2 toxin, in the range of 0–100 ng mL^−1^, with different SpyTag-FPs are shown in Fig. 3. The highest sensitivity in terms of S/B ratio was achieved with the SpyTag-mScarlet-I fusion. The SpyTag-mCherry showed the worst S/B ratio, followed by the SpyTag-TagRFP and the SpyTag-ZsYellow. These results correlated in part with the values of the calculated emission quantum yields (QYs) that were in the order: SpyTag-mScarlet-I (0.34) > SpyTag-TagRFP (0.28) > SpyTag-mCherry (0.08), except for the ZsYellow (0.022) that emits at shorter wavelengths (${\lambda }_{emi}^{max}$= 541 nm) than the others. The QY obtained for the SpyTag-ZsYellow was much lower than the respective fluorescent protein [29]. This could be due to a possible substitution of a key amino acid in the fusion protein that interacts with the chromophore and, although it provided greater ionic stability (hypsochromic shift, Supplementary Material Figure S3), it also caused a very significant decrease in the quantum yield [30].

**Fig. 4:**
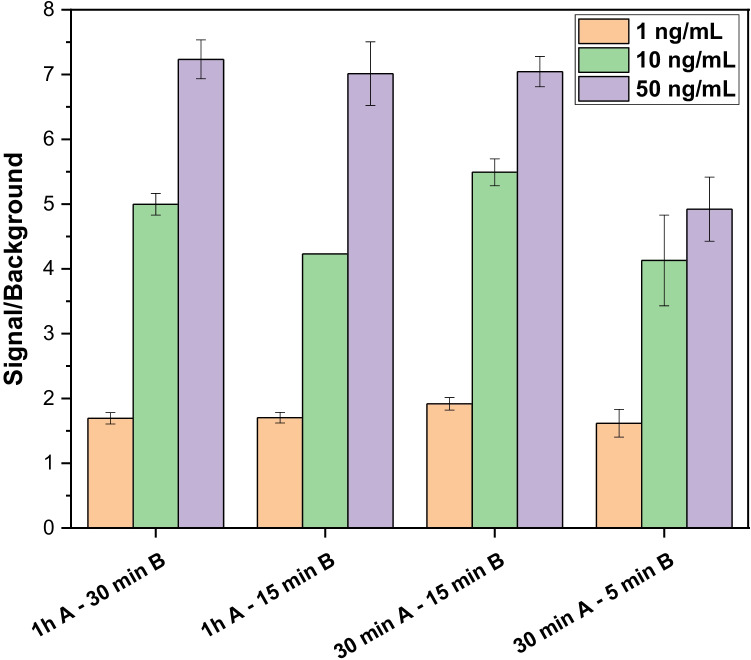
Optimisation of the incubation times: (**A**) sample and scFv-SpyCatcher; (**B**) SpyCatcher/SpyTag ligation. The signal-to-background (S/B) ratios are shown as S/B ratio means ± the standard error of the mean (*n* = 3)

Different incubation times for the interaction of the sample with the scFv-SpyCatcher (A) and for the SpyCatcher/SpyTag ligation (B) for signal generation were tested, and the results are shown in Fig. 4. Decreasing the incubation time of the sample with the antibody fragment from 1 h to 30 min did not affect the sensitivity of the assay. However, shorter sample incubation times resulted in a decrease in the signal and the S/B ratio. On the other hand, 15 min was enough for SpyTag-SpyCatcher binding to achieve the highest sensitivity. Shorter incubation times (5 min) also resulted in the ligation of the system; however, the sensitivity of the assay was lower. This demonstrates the rapid interaction between SpyTag and SpyCatcher, even if they are at low concentration [16]. Finally, an incubation time of 30 min for step A and of 15 min for B was selected for further assays. This represents a considerable decrease in the analysis time (approx. 75 min/assay) compared to previous ELISAs (approx. 150 min/assay) for the analysis of oat samples [31].

### Assay characterisation

The neutravidin-coated magnetic beads were incubated first with the bio-Fab, and after washing, followed by the addition of the sample and the scFv-SpyCatcher. After a second washing step (3 × PBS-T), the magnetic beads were incubated with the SpyTag-mScarlet-I fusion protein for signal generation.

**Fig. 5:**
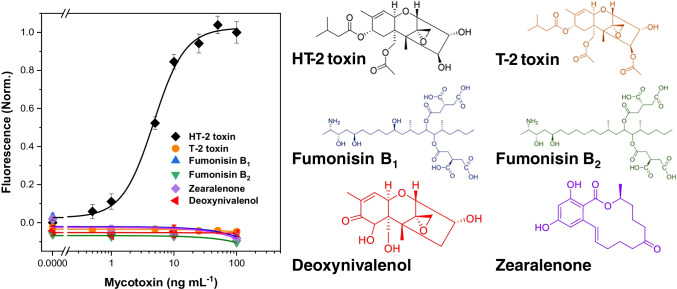
Cross-reactivity of the non-competitive immunoassay evaluated by testing different Fusarium mycotoxins (structures in the right side): HT-2 toxin (black diamonds), T-2 toxin (orange circles), fumonisin B1 (blue triangles), fumonisin B2 (green triangles), deoxynivalenol (red triangles) and zearalenone (purple diamonds). The calibration plots are expressed as normalised signal means ± the standard error of the mean (*n* = 3)

The calibration curve for the analysis of HT-2 toxin (0–100 ng mL^−1^) in the assay buffer is shown in Fig. 5. The limit of detection (LOD) was 0.24 ng mL^−1^ and the EC_50_ value was 4.8 ± 0.4 ng mL^−1^ with an inter-day relative standard deviation (RSD) of 8.5% (*n* = 3). The dynamic range (calculated as 20% and 80% of the fluorescence signal) was between 1.7 ± 0.3 and 13 ± 2 ng mL^−1^. The SpyTag-mScarlet-I has proven to be stable for more than 6 months upon storage at − 80 °C in PBS.

In order to test the cross-reactivity of the optimised method, the assay was carried out in the presence of other common toxins produced by *Fusarium* fungi species. None of the assayed mycotoxins, namely, T-2 toxin, fumonisin B_1_ and B_2_, deoxynivalenol or zearalenone cross-reacted with the antibody pair, including T-2 toxin (Fig. 5). This behaviour can be explained considering that although the primary Fab recognises both HT-2 and T-2 toxins, the secondary antibody, anti-IC HT2(10) scFv, binds selectively to the bio-Fab-HT-2 complex. Thus, the use of this approach allows a significant increase in the selectivity of the method in comparison to traditional competitive immunoassays [9, 32]. In conclusion, this assay provides a better sensitivity and selectivity, shorter analysis times and simplicity, than other assays described previously (Supplementary Material Table S2) for the analysis of HT-2 toxin.

### Sample analysis

**Fig. 6:**
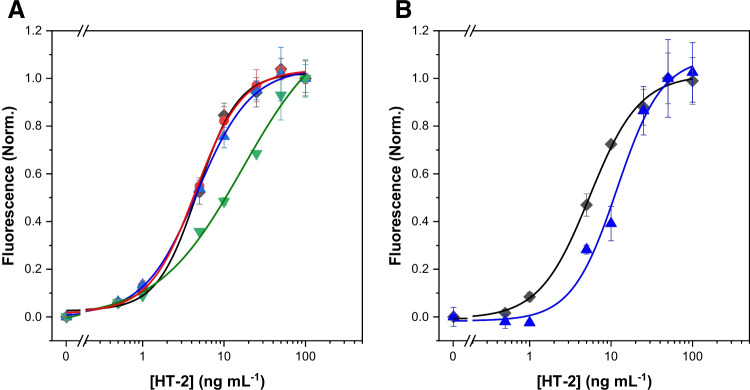
Dose–response curves for the assessment of the matrix effect in oat samples, using different detection systems: SpyTag-mScarlet-I (**A**) and SpyTag-ZsYellow (**B)**. Calibration plots were performed in assay buffer (PP Free blocking buffer in PBS) (black diamonds), in sample extracts diluted (v/v): 1:10 (red circles), 1:5 (blue triangles) and 1:3 (green triangles) in assay buffer. The results are depicted as normalised signal means ± the standard error of the mean (*n* = 3)

The developed immunoassay was applied to the analysis of real oat samples. The matrix effect was evaluated by performing calibration curves (HT-2 toxin: 0–100 ng mL^−1^) in both sample extracts and the assay buffer. Non-contaminated oat samples were extracted with MeOH:water (70:30, v/v), and after centrifugation and evaporation, the extracts were diluted at different ratios in the assay buffer (PP Free blocking buffer) to prepare matrix-matched calibration curves. The assays were carried out using the SpyTag-mScarlet-I fusion (${\lambda }_{em}^{max}:$ 593 nm) and the SpyTag-ZsYellow fluorescent protein (${\lambda }_{em}^{max}: 541\mathrm{ nm}$), for detection purposes. Standard and matrix-matched calibration plots were carried out using both fluorescent fusions proteins. When the blank oat extracts were diluted 1:5 in the assay buffer, a matrix effect was observed in the calibration plot, when the detection was carried out using the SpyTag-ZsYellow, but not with the SpyTag-mScarlet-I fusion protein (Fig. 6). This finding demonstrates the applicability of the SpyTag/SpyCatcher system for the rapid selection of the fluorescent label in immunoassay development. The matrix interference observed when using the SpyTag-ZsYellow fusion protein could be attributed to the high carotenoid content of the oats [33]. These compounds absorb light in the same region as ZsYellow [34]. A higher-fold dilution of the sample was required to avoid the matrix interference with the SpyTag-ZsYellow, resulting in much higher LODs. Therefore, the SpyTag-mScarlet-I fluorescent protein was selected for further measurements, as it minimised the matrix effect and provided lower LODs (0.6 µg kg^−1^). This value is much lower than those reported in the literature for the analysis of HT-2 in cereal samples [9–11, 14, 31] (Supplementary Material, Table S2).

The assay was applied to the analysis of an oat reference material with an HT-2-certified value of 13 ± 5 µg kg^−1^, and the results confirmed that there were not significant differences with the optimised method 13.1 µg kg^−1^ (*n* = 3, 3 assays/replicate), obtaining no significant differences at a 95% confidence level. Moreover, the oat reference material also contained T-2 toxin (7 ± 5 µg kg^−1^), but it did not affect the analysis of the HT-2 toxin due to the selectivity of the optimised method.

In parallel, the assay was applied to the analysis of a naturally contaminated oat sample and samples spiked with the toxin at concentrations in the range of 10 to 50 µg kg^−1^ (Table 1). Recoveries ranged between 93 and 104% with coefficients of variation between 4 and 15%. The results were validated by HPLC–MS/MS (HPLC–MS/MS methodology, Supplementary Material), and no significant differences (at 95% confidence level) were observed between the concentrations obtained with both methodologies. Moreover, as shown in Table 1, the concentration of HT-2 in a naturally contaminated sample was below the LOD of the HPLC–MS/MS method, but it could be analysed using the immunoassay. However, no significant differences (at 95% confidence level) were observed between the concentrations obtained with the assay and the chromatographic method in the analysis of the naturally contaminated samples spiked with 20 µg kg^−1^ of HT-2. These results demonstrate the applicability of the developed method to the analysis of HT-2 in oat at levels well below the recommended values set by the European Union [4].

**Table 1:** Analysis of different oat samples by the immunoassay (*n* = 3 replicates, 3 wells/replicate) and HPLC–MS/MS (*n* = 3)

		**Immunoassay**			**HPLC–MS/MS**		
	HT-2 concentration (µg kg^−1^)	Measured (sd) µg kg^−1^	Recovery (sd), %	CV, %	Measured (sd) µg kg^−1^	Recovery (sd), %	CV, %
1	(13 ± 5)^a^	13.1 (1.7)	101 (13.2)	13	12.5 (1.1)	96 (8.8)	9
2	10^b^	11.1 (0.9)	111 (8.8)	8	10.6 (0.9)	106 (8.6)	8
3	20^b^	20.4 (0.9)	102 (4.3)	4	20.1 (1.1)	100 (5.3)	5
4	50^b^	46.6 (4.5)	93 (8.9)	10	50.1 (3.1)	100 (6.2)	6
5	-	5.9 (0.9)	-	15	< LOD	-	-
	20^b^	26.8 (2.4)	104^c^ (9.3)	9	25.4 (0.8)	127^d^ (3.7)	3

^a^Certified concentration; ^b^spiked level; ^c^recovery considering the concentration of HT-2 toxin in the sample quantified with the optimised method; ^d^recovery in the spiked sample

## Conclusions

The SpyTag and SpyCatcher system was applied for the first time for the production and bioconjugation of recombinant proteins for the detection of mycotoxins. This is a promising tool for the development of immunoassays that allows the rapid, in situ, bioconjugation of the selective antibody with the appropriate detection system (e.g. fluorescent proteins, enzymes). In addition, there are other systems based on glue proteins (e.g. SnoopTag/SnoopCatcher) orthogonal to the SpyTag/SpyCatcher system which open up the possibility of simultaneous and selective determination of several mycotoxins in a single multiplex assay. Moreover, the SpyTag peptide and the SpyCatcher are commercially available and in principle, they could be used in combination with conventional derivatization techniques without the need for recombinant antibody technology [19].

The production of recombinant fusion proteins provides an attractive and versatile alternative to chemical conjugations, offering an effective and unlimited production system with a constant stoichiometric ratio between the fusion partners and avoiding batch-to-batch variations. Thanks to the versatility and operability of the SpyTag/SpyCatcher system, different fluorescent proteins were evaluated without the need to fuse the antibody with different fluorescent proteins. The best sensitivity for the analysis of oat samples were achieved with the SpyTag-mScarlet-I fusion protein. The bioassay has been applied to the analysis of naturally contaminated and fortified oat samples, obtaining a much lower LOD than those reported in the literature, and the results were validated analysing a certified matrix material and HPLC–MS/MS.

In summary, the use of these glue proteins for post-translational protein bioconjugation provides great flexibility for the development of immunoassays. The selected detection system genetically linked to the SpyTag or SpyCatcher can be used with any antibody labelled with the complementary part or, alternatively, the detection system can be changed easily to select the optimal one for a particular application.

## Supplementary Information

Below is the link to the electronic supplementary material.

Supplementary file1 (DOCX 815 kb)
