# Supplementary material for: Molecular super-gluing: a straightforward tool for antibody labelling and its application to mycotoxin biosensing
Source: Anal Bioanal Chem. 2022 Jan 3;414(18):5373–84. doi: 10.1007/s00216-021-03841-3 (PMC9242940; doi:10.1007/s00216-021-03841-3)
Supplement: Supplementary file 1 — Supplementary file1 (DOCX 815 kb) [file 216_2021_3841_MOESM1_ESM.docx]

**Molecular super-gluing: a straightforward tool for antibody labelling and its application to mycotoxin biosensing**

Fernando Pradanas-González^1^, Bettina Glahn-Martínez^1^, Elena Benito-Peña^1^*, Henri O. Arola^2^, Tarja K. Nevanen^2^, María C Moreno Bondi^1^*

^1^ *Department of Analytical Chemistry, Faculty of Chemistry, Complutense University of Madrid, Ciudad Universitaria s/n, 28040 Madrid, Spain*

^2^ *VTT Technical Research Centre of Finland, Tietotie 2, 02150 Espoo, Finland*

*Corresponding authors:

María Cruz Moreno-Bondi e-mail [mcmbondi@ucm.es](mailto:mcmbondi@ucm.es), Elena Benito-Peña e-mail [elenabp@ucm.es](mailto:elenabp@ucm.es)

Fernando Pradanas-González (orcid: 0000-0002-0727-7737); Bettina Glahn-Martínez (orcid: 0000-0003-0128-3113); Elena Benito-Peña (orcid: 0000-0001-5685-5559); Henri O. Arola (orcid: 0000-0002-4931-2529); Tarja K. Nevanen (orcid: 0000-0001-8964-1012); María C. Moreno-Bondi (orcid: 0000-0002-3612-0675).

**Supplementary Material**

**HPLC-MS/MS methodology 2**

**Table S13**

**Table S24**

**Figure S15**

**Figure S26**

**Figure S37**

**Figure S48**

**Figure S59**

**Figure S610**

**HPLC-MS/MS methodology**

HPLC-MS/MS validation measurements were carried out at the Institute of Food Science, Technology and Nutrition (ICTAN, Madrid, Spain). Briefly, the measurements were carried out in a high-performance chromatography instrument Agilent 1200, coupled to a Triple Quadrupole Agilent G6410B (from Agilent technologies, Santa Clara, CA, USA). Mycotoxin separation was performed with a Poroshell 120 EC C18 (3 μm x 150 mm x 2.7 mm) chromatographic column (from Agilent Technologies). The separation was accomplished under gradient conditions by using an aqueous 0.1% formic acid solution (solvent A) and MeCN supplemented with 0.1% formic acid (solvent B) at a flow rate of 0.3 mL min^-1^ (0 min, 30% B; 15 min, 80% B; 17 min, 100% B; 20 min, 100% B; 25 min, 30% B; 30 min, 30% B). The injection volume was 5 µL. The precursor ion was 447.2 m/z, and two product ions were employed in order to identify (285.1 m/z) and quantify (345.1 m/z) the HT-2 toxin.

| **Table S1**. Sequence of primers used for PCR amplifications. Red sequence corresponds to the oligonucleotide sequence of the primer used for hybridization with the DNA template and black sequence to the tails used to generate overlapping areas and add features. | |
| --- | --- |
| **Primer name** | **5´[Sequence]3´** |
| FwP_SpyTag_mS | **TGGATGCGTATAAACCGACCAAAGGTGGAGGTTCGGTGAGCAAGGGCGAGGCA** |
| RP_SpyTag_mS, RP_SpyTag_mC | **GCTCTGAAAATACAGGTTTTCCTTGTACAGCTCGTCCATGC** |
| FwP_SpyTag_mC | **TGGATGCGTATAAACCGACCAAAGGTGGAGGTTCGGTGAGCAAGGGCGAGGAG** |
| FwP_SpyTag_Y | **TGGATGCGTATAAACCGACCAAAGGTGGAGGTTCGGCTCATTCAAAGCACGGTCT** |
| RP_SpyTag_Y | **GCTCTGAAAATACAGGTTTTCGGCCAAGGCAGAAGGGAA** |
| FwP_SpyTag_T | **TGGATGCGTATAAACCGACCAAAGGTGGAGGTTCGGTGTCTAAGGGCGAAGAGCT** |
| RP_SpyTag_T | **GCTCTGAAAATACAGGTTTTCATTAAGTTTGTGCCCCAGTT** |
| FwP_SpyTag_pQE | **ACCTGTATTTTCAGAGCCATCACCACCATCATCATTAG** |
| RP_SpyTag_pQE | **CGGTTTATACGCATCCACCATCACAATATGCGCCATATGATTTATATCTCCTTCTT** |
| FwP_scFv | **CAGGTACAGCTGCAGCAGTCA** |
| RP_scFv | **GCCTGCGGCCGCACCTAG** |
| FwP_SpyCat | **TAGGTGCGGCCGCAGGCGGAGGATCCGAAGAAGATAGTGCTACCCAT** |
| RP_SpyCat | **ATGATGGCTCTGAAAATACAGGTTTTCACCTTTAGTTGCTTTGCCAT** |
| FwP_pMAL | **GAAAACCTGTATTTTCAGAGCCATCATCATCATCATCATTAG** |
| RP_pMAL | **TGACTGCTGCAGCTGTACCTGCATAATCTATGGTCCTTGTTG** |
| Sequence encoding the **mCherry fluorescent protein** was amplified with the primers **FwP_SpyTag_mC** and **RP_SpyTag_mC**, the **TagRFP fluorescent protein** with **FwP_SpyTag_T** and **RP_SpyTag_T** and the **ZsYellow fluorescent protein** with **FwP_SpyTag_Y** and **RP_SpyTag_Y**. | |

| **Table S2.** Analytical characteristics of selected methods reported in the literature for the analysis of the HT-2 toxin in cereal samples. | | | | | | |
| --- | --- | --- | --- | --- | --- | --- |
| **Sample** | **Assay format** | **Detection method** | **Analyte** | **IC_50_ or EC_50_ (µg kg^-1^)** | **LOD (µg kg^-1^)** | **Ref.** |
| Breakfast cereal (wheat), wheat and maize-based baby food | Competitive | Surface plasmon resonance (SPR) | Sum of HT-2 and T-2 toxins | 70.3 – 83.1 | 25 – 26 | [1] |
| Wheat | Competitive | Fluorescence polarisation | Sum of HT-2 and T-2 toxins | 28.8 | 8 | [2] |
| Cereals and cereal-based products | Competitive | Fluorescence polarisation | Sum of HT-2 and T-2 toxins |  | 20 – 70 | [3] |
| Wheat | Non-competitive | Time-resolved fluorescence resonance energy transfer (TR-FRET) | HT-2 toxin | 480 | 19 | [4] |
| Wheat, barley and oats | Non-competitive | ELISA | HT-2 toxin | 250 | 4 – 16 | [5] |
| Oat | Non-competitive | Fluorescence intensity | HT-2 toxin | 12 | 0.6 | This work |

1. Meneely JP, Sulyok M, Baumgartner S, Krska R, Elliott CT. A rapid optical immunoassay for the screening of T-2 and HT-2 toxin in cereals and maize-based baby food. Talanta. 2010;81:630–636. https://doi.org/10.1016/j.talanta.2009.12.055

2. Lippolis V, Pascale M, Valenzano S, Pluchinotta V, Baumgartner S, Krska R, Visconti A. A rapid fluorescence polarisation immunoassay for the determination of T-2 and HT-2 toxins in wheat. Anal Bioanal Chem. 2011;401:2561–2571. https://doi.org/10.1007/s00216-011-5379-3

3. Porricelli ACR, Lippolis V, Valenzano S, Cortese M, Suman M, Zanardi S, Pascale M. Optimisation and Validation of a Fluorescence Polarisation Immunoassay for Rapid Detection of T-2 and HT-2 Toxins in Cereals and Cereal-Based Products. Food Anal Methods. 2016;9:3310–3318. https://doi.org/10.1007/s12161-016-0527-1

4. Arola HO, Tullila A, Kiljunen H, Campbell K, Siitari H, Nevanen TK. Specific Noncompetitive Immunoassay for HT-2 Mycotoxin Detection. Anal Chem. 2016;88:2446–2452. https://doi.org/10.1021/acs.analchem.5b04591

5. Arola H, Tullila A, Nathanail A, Nevanen T. A Simple and Specific Noncompetitive ELISA Method for HT-2 Toxin Detection. Toxins. 2017;9:14 . https://doi.org/10.3390/toxins9040145


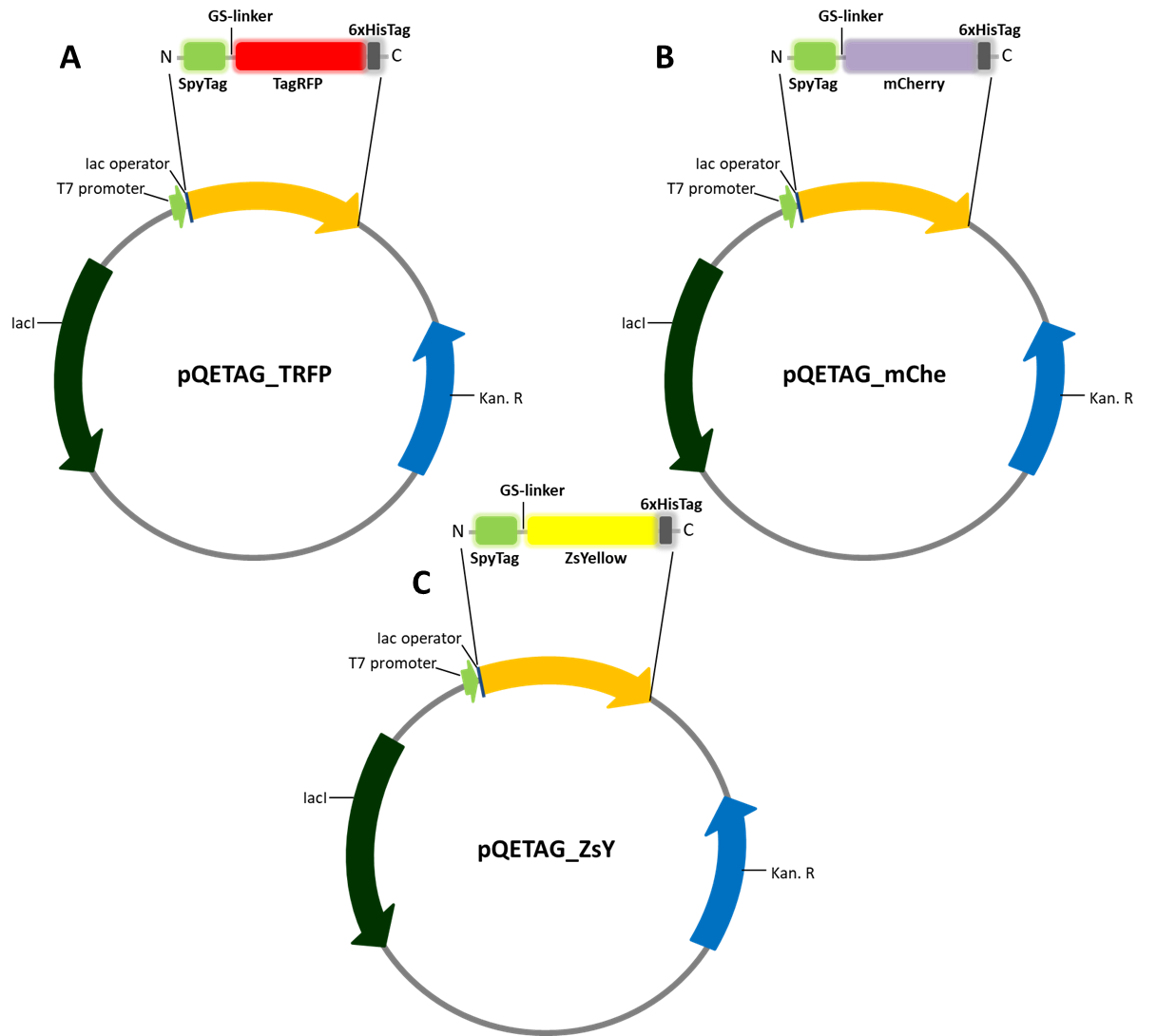


**Figure S1.** Scheme of the fluorescent fusion protein constructs and main characteristics of expression vector used for the production of recombinant proteins in *E. coli* BL-21 (DE3) pLysS. A: SpyTag-TagRFP in the vector pQETAG_TRFP; B: SpyTag-mCherry in pQETAG_mChe; C: SpyTag-ZsYellow in pQETAG_ZsY.


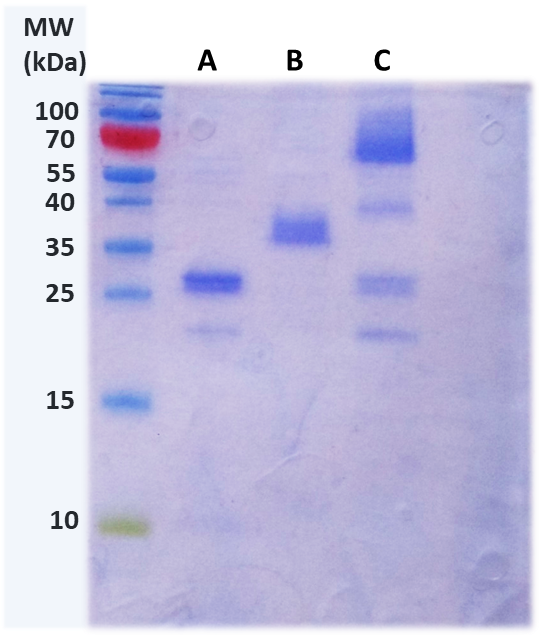


**Figure S2.** Verification of the posttranslational bioconjugation by SDS-PAGE. A: SpyTag-mScarlet-I (30 kDa); B: anti-IC HT-2 (10) acFv-SpyCatcher (38 kDa); C: bioconjugation of fusion proteins (70 kDa) by formation of a spontaneous isopeptide bond between SpyTag and SpyCatcher (after 30 min of incubation in a mole ratio 1:1 at room temperature and with shaking).


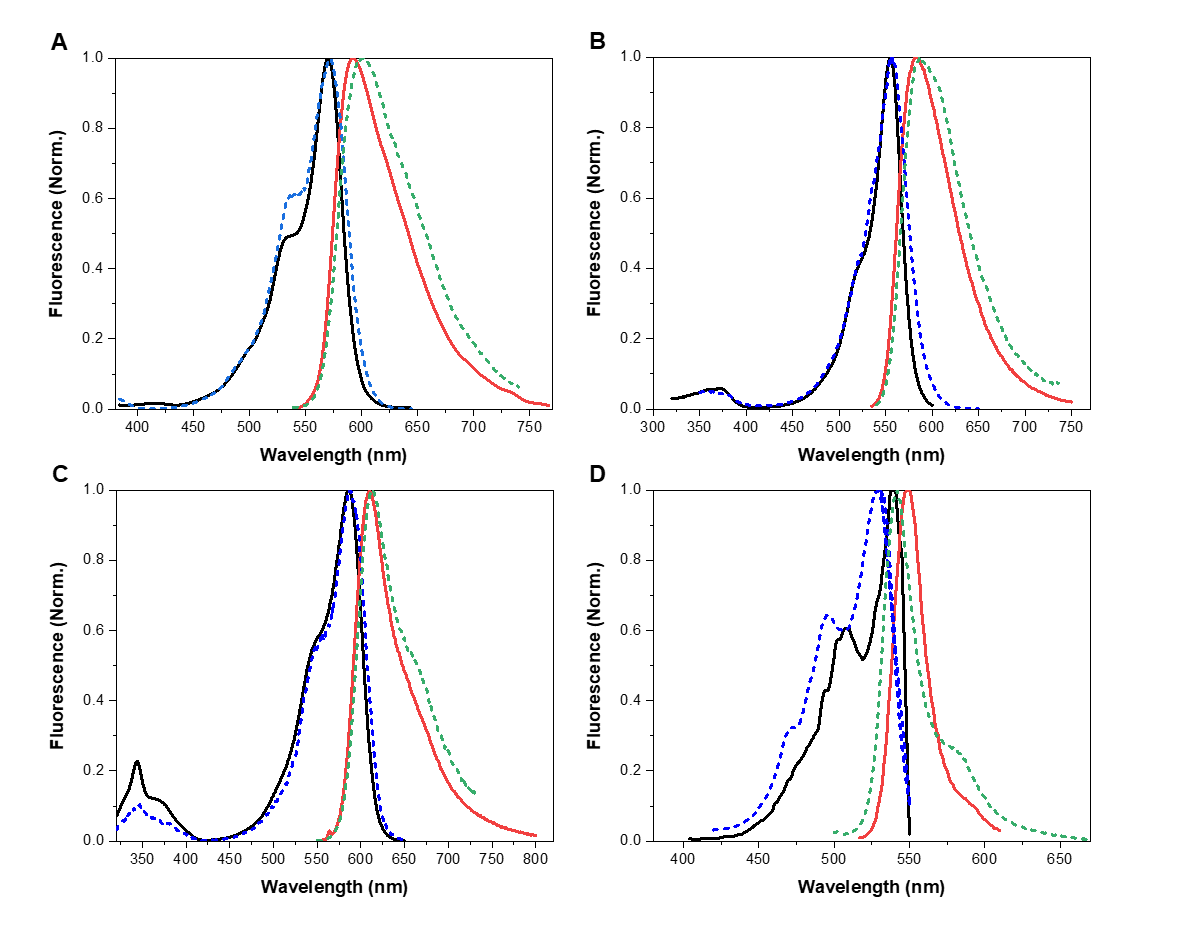


**Figure S3.** Theorical excitation (solid black line) and emission (solid red line) spectra of the isolated fluorescent proteins and excitation (dash blue line) and emission (dash green line) spectra of the SpyTag-FP fusion proteins: SpyTag-mScarlet-I (A), SpyTag-TagRFP (B), SpyTag-mCherry (C) and SpyTag-ZsYellow (D).


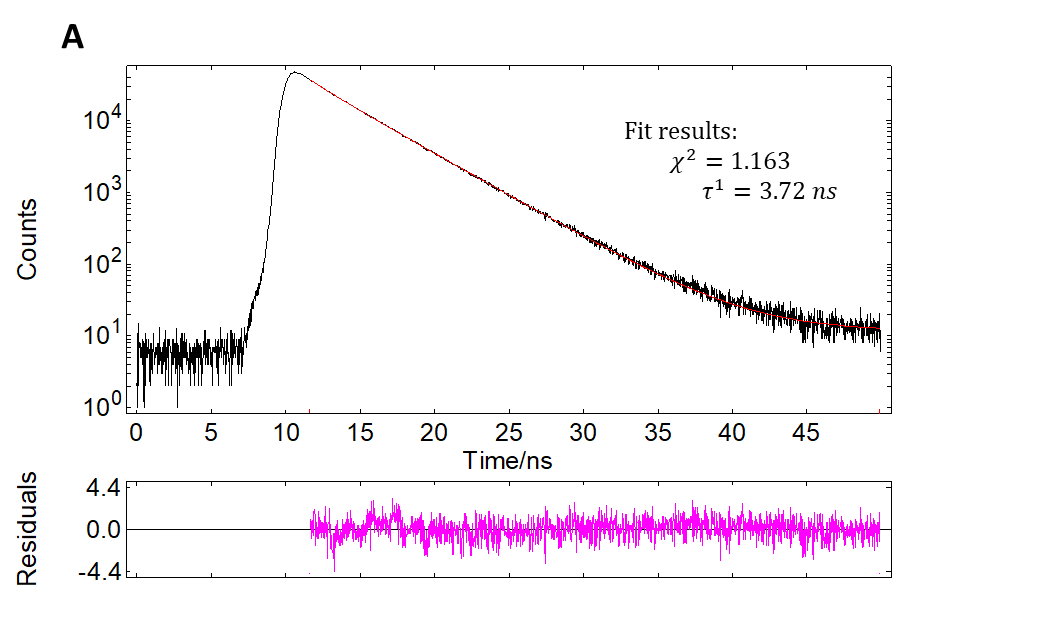

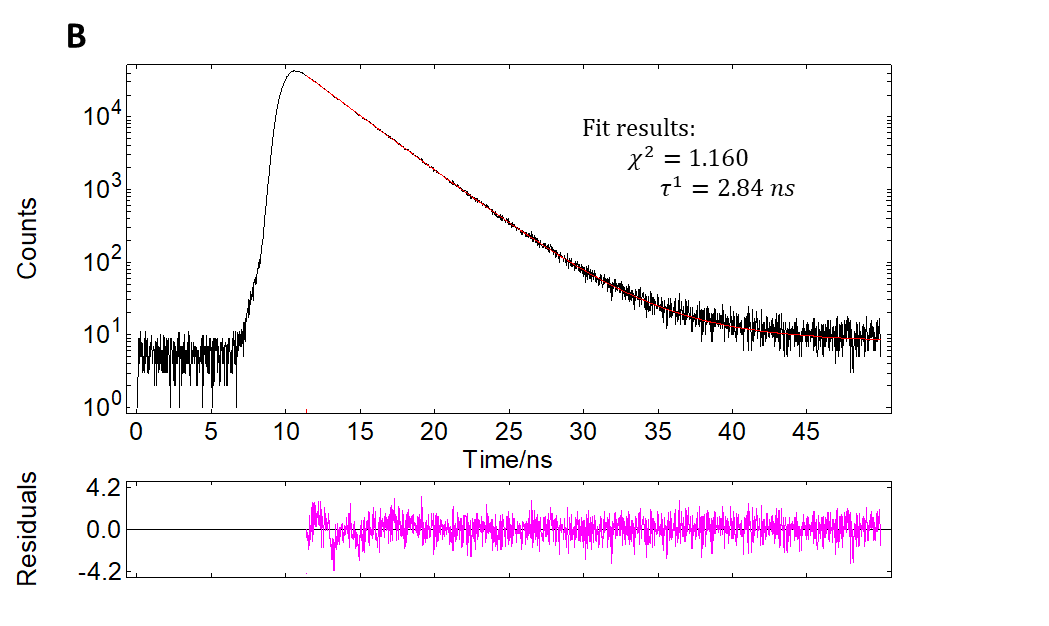


**
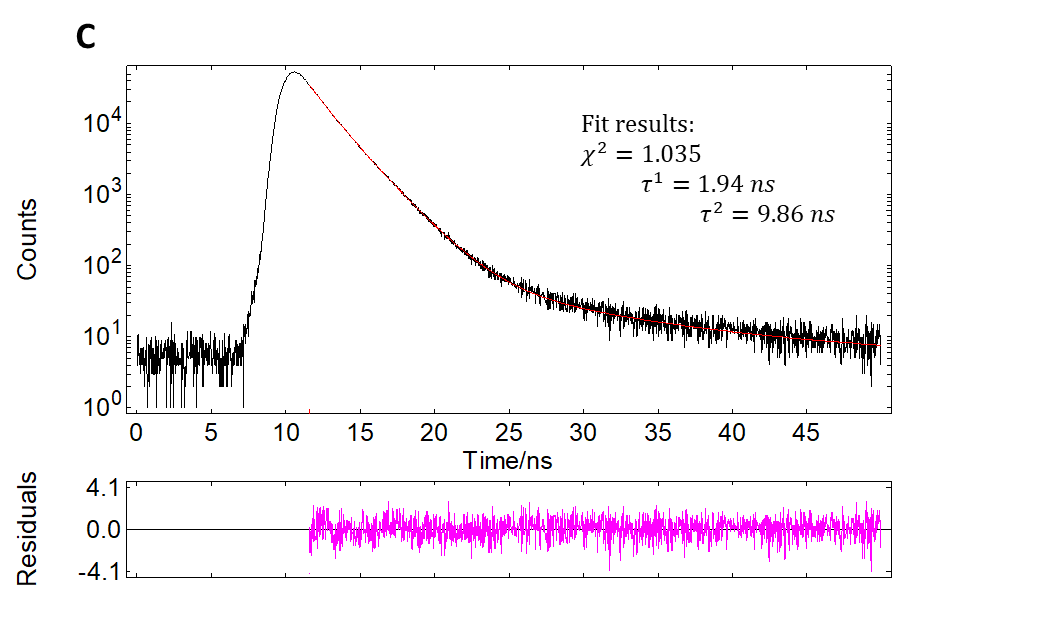
**

**
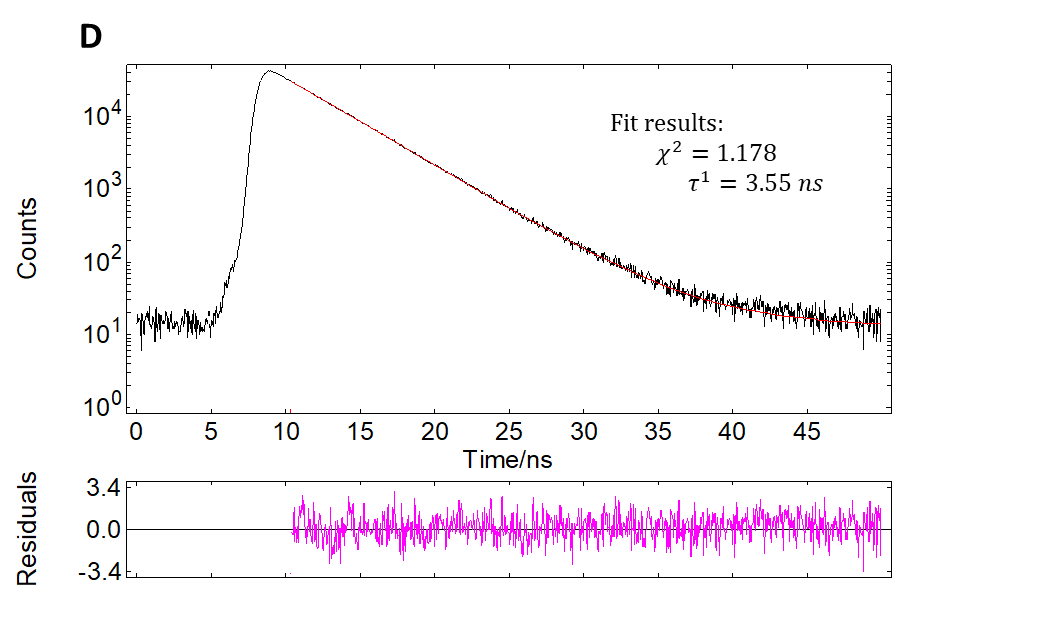
**

**Figure S4.** SpyTag-mScarlet-I (A), SpyTag-TagRFP (B), SpyTag-mCherry (C) and SpyTag-ZsYellow (D) fusion proteins emission decays measured in PBS. Excitation was performed with a 463 nm < 1 ns laser diode pulsed at 500 KHz through a 460 nm band-pass interference filter- Emission was monitored at 593 nm (A) 584 nm (B), 611 nm (C) and 541 nm (D) through a 500 nm blazed double monochromator, with a Hamamatsu R928P photomultiplier tube. In all cases, 40000 counts were collected at the peak channel.

**Figure S5.** Optimisation of the assay buffer and the support for the immobilisation of the biotinylated Fab-anti-HT-2. All experiments were assayed with 500 ng of biotinylated Fab (60 μL) anti-HT-2 and 700 ng of anti-IC HT-2 (10) scFv-SpyCatcher (60 μL). Green bars correspond to the fluorescence signal for 5 ng ml^-1^ of HT-2 toxin and blue bars to the signal for the negative control (0 ng ml^-1^ of HT-2 toxin). The optimisation was carried out with the SpyTag-TagRFP fusion protein. The results are shown as fluorescence signal or signal-to-background ratio means ± the standard error of the mean (n=3).

**Figure S6.** Optimisation of biotinylated anti-HT-2 (10) Fab and anti-IC HT-2 (10) scFv-SpyCatcher amounts. Green bars correspond to the fluorescence signal for 5 ng ml^-1^ of HT-2 toxin and blue bars the signal for the negative control (0 ng ml^-1^ of HT-2 toxin). The optimisation was carried out with the SpyTag-TagRFP fusion protein. The results are depicted as fluorescence signal or signal-to-background ratio means ± the standard error of the mean (n=3).
